# Race and Sex Disparities in Metabolic/Bariatric Surgery over 20 Years: A Cohort Study

**DOI:** 10.1097/AS9.0000000000000540

**Published:** 2025-01-15

**Authors:** Benjamin Grobman, Liyun He, Zhou Lan, Abdelrahman Nimeri, Caroline Apovian, Alexander Turchin

**Affiliations:** From the * Harvard Medical School, Boston, MA; † Division of Endocrinology, Brigham and Women’s Hospital, Boston, MA; ‡ Department of Endocrinology, Peking Union Medical College Hospital, Beijing, China; § Center for Clinical Investigation, Brigham and Women’s Hospital, Boston, MA; ∥ Division of Gastrointestinal and General Surgery, Brigham and Women’s Hospital, Boston, MA.

**Keywords:** bariatric surgery, disparities, natural language processing

## Abstract

**Objectives:**

To determine how rates of discussion of metabolic and bariatric surgery (MBS) between patients with class 2 obesity and higher and their healthcare providers as well as subsequent progression to MBS have varied by race and sex over the last 20 years.

**Background:**

Obesity is a growing public health crisis. MBS remains the most effective treatment for long-term management of obesity.

**Methods:**

Retrospective cohort study of electronic health records from a large tertiary academic health system using a previously validated natural language processing algorithm. The primary outcomes were discussion of MBS between eligible patients and providers and progression to surgery. Multivariable logistic regression was used to determine the association between sociodemographic factors, time, and discussion and receipt of MBS.

**Results:**

Among 122,487 patients included in the analysis, 11,094 (9.1%) patients discussed MBS with their providers. Of these, 1348 (12.2%) progressed to MBS. Between 2000 and 2020 the annual fraction of patients who had a discussion of MBS with their providers rose from 3.2% to 10.0% (*P* < 0.001). Black people were equally likely to discuss MBS with providers as non-Black people (9.5 vs 9.0%, *P* = 0.09) but were less likely to progress to MBS (8.4 vs 12.6%, *P* < 0.001). These disparities in progression narrowed over the study period (*P* = 0.044). Men were less likely than women to both discuss (8.1% vs 9.6%, *P* < 0.001) and receive MBS (7.6% vs 14.6%, *P* < 0.001), and these disparities increased over the study period.

**Conclusions:**

Interventions to reduce racial disparities in utilization of MBS should focus on progression from discussion to MBS. Interventions to increase the uptake of MBS among men are required across the care cascade.

## INTRODUCTION

Metabolic and bariatric surgery (MBS) remains the most effective long-term treatment for severe obesity and type 2 diabetes.^1^ Rates of obesity continue to rise in the United States, reaching 41.95% in 2017 to 2020.^2^ Rates of obesity are particularly high among Black Americans: in this same time period, the age-adjusted prevalence of obesity among Black adults was 49.9%.^2^ Black Americans also have high rates of obesity-related comorbidities such as diabetes: an estimated 17.4% of Black Americans have diabetes, compared to 13.6% of White Americans.^3^

Despite these disparities, previous research has established that Black patients are less likely to undergo MBS than patients with obesity from other racial and ethnic groups.^4^ Additionally, it has been shown that Black patients are less likely to successfully lose weight after MBS.^5^ The reasons for this disparity are multifactorial, and prior research has shown that Black patients often have worse postoperative outcomes after MBS.^6–8^

Prior research has also shown that male patients are less likely to undergo MBS than female patients.^9^ Despite a similar prevalence of obesity among males and females, 80% of patients who undergo MBS are female.^9,10^ These sex differences are particularly pronounced among low-income, Black, and Hispanic patients.^10^ While these race and sex disparities are well-established, less is known regarding the components of the care cascade that led to Black and male patients not receiving MBS. A previous survey-based study showed that among patients who actively seek MBS, there are similar rates of progression to MBS across racial and ethnic groups.^11^ However, population-based studies on this issue are lacking. Additionally, no prior studies have examined disparities in the MBS care cascade among patients who may benefit from MBS regardless of whether they are actively seeking MBS. Finally, it is not known how disparities in the MBS care cascade have changed over time.

In a previous study, we used natural language processing (NLP) to examine electronic health records (EHRs) on a population scale to determine how often providers discussed MBS with eligible patients. We reported that patients who discussed MBS with providers were more likely to undergo MBS and were more likely to lose weight than those who did not discuss MBS.^12^ It also showed that despite the importance of patient-provider discussions of MBS, only 8.7% of potentially eligible MBS patients had patient-provider discussions about MBS.^12^ The present study builds on those results to assess racial and sex disparities across the MBS care cascade and to examine how these disparities have evolved over time.

## METHODS

### Study Design

We conducted a retrospective cohort study among patients with class 2 obesity and higher to investigate changes in racial and sex disparities in patient-provider discussion of MBS and progression to MBS over time.

### Study Cohort

We retrospectively studied EHR data of adults (aged ≥18 and <65 years) with class 2 obesity and higher who were followed in primary care practices affiliated with Mass General Brigham between January 1, 2000, and December 31, 2022 (2 years after the final study entry). Patients were eligible if they had body mass index (BMI) ≥35 kg/m^2^ and <80 kg/m^2^ on 2 consecutive measurements. Patients were excluded if they had (1) prior MBS or (2) significant comorbidities that would likely be a contraindication to MBS, including heart failure, coronary artery disease, chronic lung disease, or cancer requiring hospitalization within the previous 12 months, drug/alcohol use disorder, or dementia/cognitive impairment. Patients were also excluded if they had missing demographic information (legal sex or median household income by zip code) or did not have an encounter with their primary care provider over the year following study entry (to make possible analysis of patient-provider discussions of MBS).

### Study Measurements

A patient was entered into the study on the last of the following dates: (1) first primary care note (to ensure availability of baseline clinical characteristics, as patients who only receive specialist care at the study institution are less likely to have their medical history comprehensively documented in the EHR); (2) 18th birthday; (3) date when they became eligible for MBS according to the criteria listed above; or (4) January 1, 2000. Patients could enter the study until December 31, 2020, to allow sufficient time for outcome assessment. We evaluated whether the patient had (1) a discussion of MBS with their provider documented in EHR notes during 1 year after study entry; and (2) received MBS during 2 years after study entry. Patient-provider discussion of MBS was ascertained based on documentation in EHR notes using a previously validated NLP tool^12^ based on the publicly available Canary NLP platform.^13^ Receipt of MBS was ascertained based on common procedural terminology codes in EHR. Information on baseline patient characteristics and study outcomes was obtained from the EHR at Mass General Brigham. This study was approved by the Mass General Brigham institutional review board, and the requirement for informed consent was waived.

### Statistical Analysis

An individual patient served as the unit of analysis. Univariate comparisons were conducted using two-sample *t* tests and one-way ANOVA. The association between demographic factors and discussion and receipt of MBS was determined using logistic regression models. Models were adjusted for age, year of study entry, medical comorbidities, education, insurance, and for clustering within primary care providers. To determine whether discussions and receipt of surgery changed over time, the Cochran-Armitage test was used. Logistic regression was used to determine whether disparities in discussions and receipt of surgery (based on race and gender) changed over time, by comparing slopes using a Wald test. Cross-sectional analyses were performed using SAS version 9.4 (SAS Institute Inc., Cary, NC), and time-trend analyses were performed using R (version 4.2.0).

## RESULTS

### Baseline Characteristics

**FIGURE 1.**
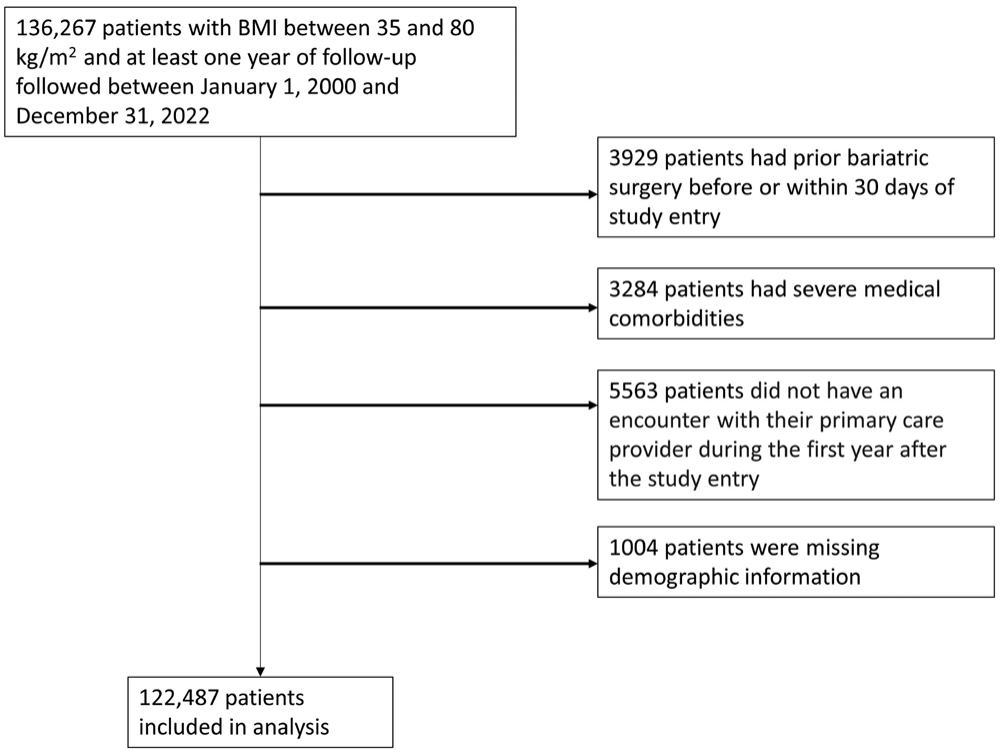
Study flow diagram.

We identified a total of 130,704 adults with class 2 obesity and higher, aged 18 to 64 years followed at Mass General Brigham between 2000 and 2020. After excluding patients who had prior MBS, likely contraindications to MBS, and individuals with missing demographic data, 122,487 patients were included in the analysis (Fig. 1). Their median age was 45.0 and their median BMI was 37.3 kg/m^2^. Among these patients, 75,557 (61.7%) were women, 91,971 (75.1%) were White, 11,881 (10.2%) were Black, 6179 (5.1%) were Hispanic, and 1751 (1.4%) were Asian (Table 1). More females as compared to males had at least some college education (43.8% vs 37.5%, *P* < 0.001). Females were less likely than males to have diabetes (6.6% vs 8.7%, *P* < 0.001). Mean BMI was higher among females as compared to male participants (39.3 vs 38.9, *P* < 0.001). Prevalence of at least some college education was similar between Black and non-Black participants (41.4% vs 41.4%, *P* = 0.9). Rates of diabetes were higher among Black participants (10.0% vs 7.1%, *P* < 0.001). Mean BMI was higher among Black participants as compared to non-Black participants (39.4 vs. 39.1, *P* < 0.001).

**TABLE 1.** Baseline Characteristics of Study Patients

	All Patients	Patients Who Discussed MBS
Age (y), mean (SD)	43.6 (12.7)	43.7 (12.2)
Female, n (%)	75,557 (61.7)	7287 (65.7)
White race, n (%)	91971 (75.1)	8104 (73.1)
Black race, n (%)	11881 (9.7)	1127 (10.2)
Government Insurance, n (%)	36291 (29.6)	3512 (31.7)
Baseline BMI, kg/m^2^ mean (SD)	39.2 (4.5)	43.0 (7.1)
Hypertension, n (%)	29871 (24.4)	2750 (24.8)
Diabetes, n (%)	9078 (7.4)	1287 (11.6)
Charlson Comorbidity Index, mean (SD)	1.0 (1.8)	1.1 (1.9)
English as primary language, n (%)	114085 (93.1)	10349 (93.3)
Married, n (%)	61594 (50.3)	5374 (48.4)
Median household income by zip code ($1000), mean (SD)	89.9 (31.6)	88.2 (31.5)

### MBS Discussions

**FIGURE 2.**
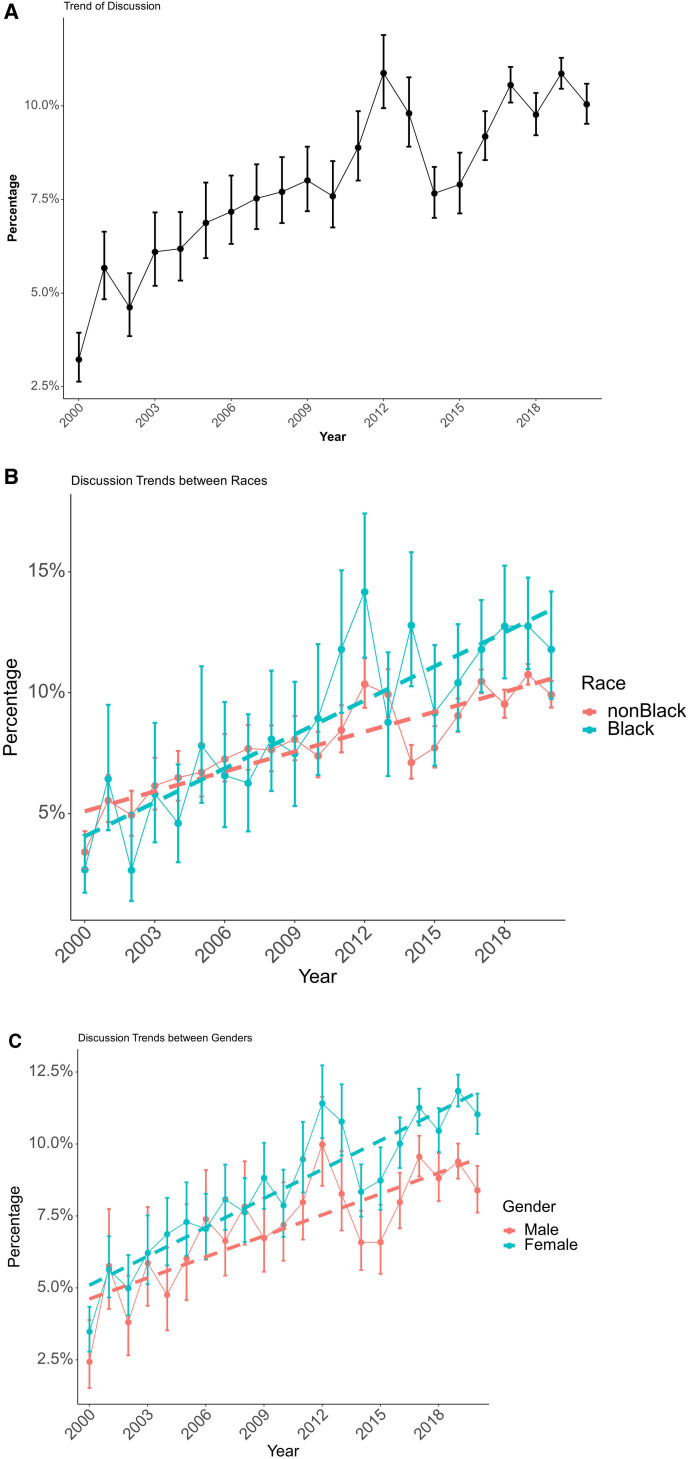
A, Rates of discussion of MBS over time. Vertical lines represent 95% confidence intervals. B, Rates of MBS discussion by race over time. Vertical lines represent 95% confidence intervals. Dashed lines represent trends over time. C, Rates of MBS discussion by sex over time. Vertical lines represent 95% confidence intervals. Dashed lines represent trends over time.

Over the study period, 11,094 (9.1%) patients discussed MBS with their providers. Between 2000 and 2020, the number of patients who had a discussion of MBS with their providers rose from 3.2% to 10.0% (Fig. 2A).

Women had MBS discussions more frequently than men (9.6% vs 8.1%, *P* < 0.001). In a multivariable analysis adjusted for patient demographics, year of study entry, BMI, and comorbidities (Table 2), women were more likely than men to discuss MBS with their providers (OR = 1.17; 95% CI = 1.12–1.22). The difference in patient-provider MBS discussions between women and men increased (Fig. 2C) on average by 0.9% per year over the study period (*P* = 0.024). Those who discussed MBS had higher mean BMI than those who did not discuss MBS (43.0 vs 39.2) with higher odds of discussion on multivariable analysis (OR = 1.137; 95% CI = 1.133–1.140).

**TABLE 2.** Patient Characteristics and Probability of MBS Discussion: Multivariable Analysis

Variable	Odds Ratio (95% CI)	*P* value
Age, years	0.999 (0.997–1.000)	0.114
Female sex	1.170 (1.120–1.222)	<0.0001
Black race (vs all other races)	1.028 (0.958–1.103)	0.445
Government insurance (vs private insurance)	1.084 (1.033–1.136)	0.001
Median household income by zip code, per $10,000 increase	1.001 (0.994–1.008)	0.848
Some college education (vs less than college education)	1.131 (1.084–1.181)	<0.0001
Primary language other than English	0.999 (0.997–1.000)	<0.0001
BMI, kg/m^2^	1.137 (1.133–1.140)	<0.0001
Charlson Comorbidity Index	1.028 (1.015–1.041)	<0.0001
Hypertension	1.017 (0.969–1.069)	0.495
Diabetes	1.596 (1.478–1.724)	<0.0001
Study entry after 2010 (vs study entry before 2010)	1.919 (1.815–2.028)	<0.0001

Black and non-Black patients (Fig. 2B) discussed MBS with their providers at similar frequencies (9.5% vs 9.0%; *P* = 0.09). In a multivariable analysis (Table 2), there was no significant difference in MBS discussion rates between Black and non-Black patients (OR = 1.03; 95% CI = 0.96–1.10). Over the study period, the rates of patient-provider discussion increased by 2.3% more annually for Black compared to non-Black patients (*P* < 0.001). We also analyzed the specialty with which participants first discussed MBS: 8215 participants (74.0%) first discussed MBS with primary care physicians, 226 participants (2.0%) first discussed MBS with endocrinologists, 131 participants (1.6%) first discussed MBS with cardiologists, and 2522 (22.7) participants first discussed MBS with physicians from other specialties.

### Progression to MBS

**FIGURE 3.**
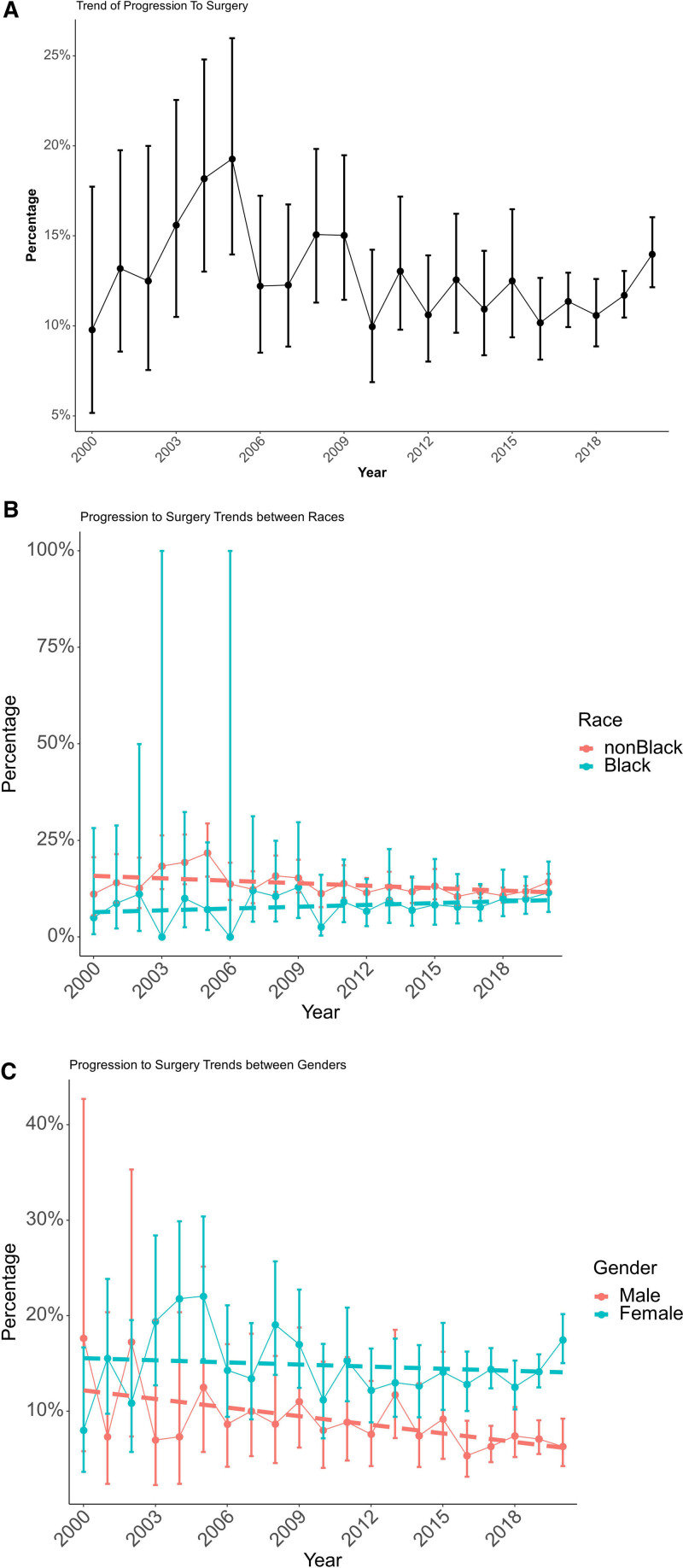
A, Rates of progression to MBS over time. Vertical lines represent 95% confidence intervals. B, Progression to surgery after MBS discussion by race over time. Vertical lines represent 95% confidence intervals. Dashed lines represent trends over time. C, Progression to surgery after MBS discussion by sex over time. Vertical lines represent 95% confidence intervals. Dashed lines represent trends over time.

Among 11,094 study patients who had a discussion of MBS, 1348 (12.2%) progressed to MBS. More women than men progressed to MBS (14.6% vs 7.6%; *P* < 0.001). The rate of progression to MBS stayed mostly unchanged over the study period (Fig. 3A). In a multivariable analysis adjusted for patient demographics, year of study entry, BMI, and comorbidities (Table 3), women were more likely to proceed to MBS after discussing it (OR = 2.06; 95% CI = 1.79–2.37). The difference in MBS progression rates between women and men increased over time (Fig. 3C) by 2.7% per year (*P* = 0.037). Fewer Black than non-Black patients proceeded to MBS (8.4% vs 12.6%; *P* < 0.001). In multivariable analysis (Table 3), Black patients were less likely to proceed to MBS than patients from other racial and ethnic groups (OR = 0.56; 95% CI = 0.45–0.70). The difference in rates of progression to MBS between Black and non-Black patients decreased over time (Fig. 3B) by 4.4% annually (*P* = 0.044). Those with higher BMIs were more likely to progress to MBS than those with lower BMIs (OR = 1.081; 95% CI = 1.011–1.026) (*P* < 0.001) (Table 3). During the year 2000 (the first year examined) 21 out of 2851 patients who discussed MBS later had MBS (0.74%). In 2020, 311 out of 11,862 patients who discussed MBS later had MBS (2.55%).

**TABLE 3.** Patient Characteristics and Probability of Progression to MBS: Multivariable Analysis

Variable	Odds Ratio (95% CI)	*P* value
Age, years	0.994 (0.989–0.999)	0.023
Female sex	2.062 (1.791–2.374)	<0.0001
Black race (vs. all other races)	0.559 (0.446–0.702)	<0.0001
Government insurance (vs. private insurance)	0.979 (0.858–1.119)	0.758
Median household income by zip code, per $10,000 increase	0.990 (0.971–1.010)	0.336
Some college education (vs less than college education)	1.403 (1.245–1.582)	<0.0001
Primary language other than English	0.984 (0.774–1.250)	0.892
BMI, kg/m^2^	1.018 (1.011–1.026)	<0.0001
Charlson Comorbidity Index	1.039 (1.003–1.076)	0.034
Hypertension	0.574 (0.490–0.673)	<0.0001
Diabetes	1.062 (0.866–1.302)	0.564
Study entry after 2010 (vs study entry before 2010)	0.724 (0.621–0.843)	<0.0001

## DISCUSSION

In this large population-based study of over 100,000 adults potentially eligible for MBS, we showed that there are significant disparities in both the discussion and receipt of MBS. Specifically, Black people were less likely to receive MBS, and men were less likely than women to both discuss and receive MBS. While racial disparities have decreased slightly over time, sex disparities in receipt of MBS have grown significantly over the past 20 years.

Our finding that Black people are less likely to receive MBS aligns with prior research which has shown that despite higher rates of obesity, Black Americans are less likely to receive MBS.^4^ Importantly, a novel finding of this study is that racial disparities in MBS are predominantly represented in progression to MBS rather than discussion of MBS. This indicates that interventions to increase the uptake of MBS should be focused on improving discussion quality and progression through the care cascade for Black people. Prior research has shown that Black patients are less likely to accept recommendations for surgical procedures.^14–16^ Possible reasons for this observation include implicit provider bias, higher rates of medical mistrust, and lower levels of health literacy.^16,17^ A prior study to reduce disparities in time to lung cancer surgery successfully used tools such as EHR notifications, patient navigators, and physician monitoring of ongoing disparities in surgical utilization.^18^ Given our findings of decreased receipt of appropriate care among Black patients eligible for MBS, a similar approach may be valuable in improving outcomes for this growing population.

The present study found that sex disparities in MBS established by previously published investigations^9^ have continued to grow throughout the last 20 years—despite the well-established similar effectiveness of MBS among men and women.^19^ This gap was pervasive across the MBS care cascade, including both patient-provider discussions of MBS and subsequent progression to surgery. Several factors may be contributing to this important disparity. On the one hand, it has previously been shown that men are less likely to engage with the healthcare system and present later in their disease courses than female patients.^9,20,21^ Furthermore, men may face lower social pressure to lose weight than women,^22^ and fewer men identify themselves as overweight.^23^ Therefore, proactive public health interventions may be required to encourage men with obesity to engage with necessary health care services.

Encouragingly, this study found that rates of discussion and utilization of MBS have increased over time. This increase in discussion rates mirrors the increased safety of MBS procedures over the past 20 years. A 2020 paper showed that in 1998 (2 years before the beginning of this study), rates of complication after MBS were 11.7% with 1% mortality after MBS, while in 2016 complications was 1.4% and mortality after surgery was 0.04%.^24^ However, recent research has been more equivocal with regard to utilization of MBS, with studies showing that MBS use increased in the early 2000s before plateauing in the 2010s.^25,26^ Although we found increasing rates of discussion, including in the most recent time period analyzed, given the advent of new antiobesity medications such as glucagon-like peptide 1 receptor agonists, future studies will be required to understand how the changing obesity pharmacotherapy landscape impacts providers’ discussions of MBS.^27^

Despite this increasing rate of discussion, we found that the conversion of discussion to receipt of MBS has remained largely unchanged throughout the study period. This result is concerning given rising rates of obesity in the United States and improved safety of MBS.^26,28^ Prior research on conditions such as cancer has shown that even when surgery is recommended, patients may not receive necessary surgery and that this issue is particularly significant among Black patients.^29^ Our findings indicate that further studies are necessary to better understand the content of providers’ discussions with patients regarding MBS. Of note, the majority of participants in this study first discussed MBS with primary care physicians, indicating that these physicians should be the primary target of interventions meant to increase MBS uptake. Additionally, future studies should examine patient barriers to receiving MBS once surgery has been recommended.

The detailed examination of the critical components of the patient care cascade—patient-provider discussion of MBS and subsequent progression to surgery—would have been impossible without the utilization of artificial intelligence technology—NLP. The NLP software made it possible to analyze over 2 million EHR provider notes in a matter of hours. As a result, this study was able to localize the origins of racial and sex disparities in MBS, facilitating the identification of potential targets for future interventions. This result is a testament to the potential of NLP—a quickly growing field^30^—as a tool in health services and outcomes research.

This study has multiple strengths. The sample size (over 100,000 patients) allowed for a powerful analysis of disparities in the MBS care cascade among sociodemographic subgroups and over time. Additionally, this is the first study to analyze the components of the MBS care cascade giving rise to disparities. Furthermore, the analysis of trends in disparities over time is unique and can inform further interventions to improve equity in MBS utilization. Finally, our manuscript used rigorous analytical methods, including hierarchical regression models, which adjusted for the clustering of patients within individual providers to minimize the impact of individual providers’ care practices.

This study also has several limitations. Our analysis was restricted to a single health system in Massachusetts and therefore may not be representative of the entire US population. While we did analyze whether patients had private or government insurance, which may serve as a proxy for insurance quality, we were not able to analyze whether patient’s insurance covered MBS. We did not have access to information on whether providers discussed other weight loss modalities such as medication or lifestyle changes with patients. Additionally, we were not able to analyze the character of MBS discussions (eg, whether the discussion of MBS was a positive or negative one, and whether it was initiated by the provider or the patient), only whether MBS was discussed. We also did not have information on the length of discussions, which may be important as prior research has shown that longer discussions (as reflected by more extensive documentation) are associated with optimal healthcare outcomes.^31^ Finally, some of the potentially important provider characteristics (eg, race and sex concordance with the patient) were not available for analysis.

This large population-based study showed that there are significant disparities in utilization of MBS by race and sex. Given the rising burden of obesity in the United States, providers and policymakers will need to intervene to ensure that people from all backgrounds have appropriate access to the expanding landscape of treatment for obesity. Additionally, this study reinforces the pervasive nature of racial health disparities in the United States health care system and serves as an indicator of significant improvement in the provision of equitable medical and surgical care.
